# The Effect of Employment on Delinquent Behavior Among Youth in Hidden Situation

**DOI:** 10.3389/fpsyt.2019.00229

**Published:** 2019-04-23

**Authors:** Gloria Hongyee Chan

**Affiliations:** City University of Hong Kong, Kowloon, Hong Kong

**Keywords:** youth, hidden, employment, delinquent behavior, social withdrawal

## Abstract

This study examines the effect of employment on delinquent behavior among young people in “hidden situations”. Both quantitative and qualitative methods were used, and 588 young people in these hidden situations were investigated. Results showed that some of them had employment; their employment status constituted a mediating effect on preventing them from being involved in delinquent behavior. Also, participants who had work explained the reasons for not being involved in delinquent behavior. These results reflect that whether young people in hidden situation involve in delinquent behavior depends on their employment rather than their hidden behavior itself. The implication is that practitioners should respect the youths’ self-preferred choice of employment and even advocate a revision of the definition of employment in Hong Kong.

## Introduction

Traditionally, it is stated that employment and delinquent behavior are related (e.g., 1–3). Stability in work can reduce young people’s delinquent behavior (e.g., 4, 5). Similar results were also found among high-risk youth (6, 7) and people with criminal records (8, 9). For youth in hidden situations, it appears to be possible that they have a high likelihood to engage in delinquent behavior such as drug-taking and drug-trafficking (10) because of their employment situations such as NEETs (i.e., “not in employment, education, or training”), freeters (i.e., “youth floating between dead-end, part-time jobs”) (11, 16), three-lows (i.e., having low education level, skill level, and motivation level) (12), failing to fit into the societal standards for education or career (13, 14), and being reluctant to engage into society (15). Also, from a clinical perspective, under limited social support, youth in prolonged hidden situations are prone to experiencing mental health issues such as sense of loneliness and low self-esteem (16, 17), and even a gradual loss of social skills and initiation to build social relationships, which further hinder their social connections and engagement in school or work (18). However, as society changes, flexibility in work arrangements is promoted and the Internet is used as a platform for handling work arrangements; this leads to the mushrooming of non-typical forms of employment, such as freelance, home-based work and Internet-based work (19–21). The increasing prevalence of such forms of work definitely triggers a rethinking of whether the traditional concept of employment still applies in current society. According to the study conducted by Chan (22) on youth in hidden situations in Hong Kong, these youth do have employment, which is one of the significant life transitions predicting a decreased likelihood delinquent behavior. This demonstrates that these youth are not jobless, only that their jobs do not match the official definitions of employment that requires a “formal job attachment” (23) and an “outside workplace” (24: 4). In this sense, youth in hidden situations are not necessarily delinquent-prone in light of their employment situation. Against this backdrop, this study seeks to further explore these youths’ nature of employment, and how their employment influences their engagement in delinquent behavior in the local context. It is expected that the study will not only help uncover the real situation of these youth in terms of their employment and its effects on their delinquent behavior but will also generate implications regarding how the concept of employment can be understood in contemporary society.

To provide support for the research aims, in the following, literature about the relationship between employment and delinquent behavior will first be reviewed.

### Employment and Delinquent Behavior

While deviant behavior refers to behavior that violates societal norms and behavioral standards, or triggers negative reactions from others (25), delinquent behavior is referred to as law-breaking behavior (26). Some behavior can be deviant but not law-breaking, while some behavior can be both deviant and law-breaking (27). In other words, deviant behavior and delinquent behavior are conceptually different but, at the same time, have overlaps.

Reviewing existing literature, there are theories supporting the significant negative relationship between employment and delinquent behavior [e.g., Refs. (28–31)]. To begin with, according to General Strain Theory (28, 29), strain (e.g., lack of financial resources) serves as a state of discomfort that triggers delinquent behavior. In this sense, employment will act as a legitimate “conventional opportunity structure” for youth to earn money, thus reducing the pressure to turn to delinquent behavior (32, 33, 301). Next, according to Routine Activities Theory (30), it is posited that environmental contexts (e.g., presence of guardianship, available opportunities for committing delinquent behavior) affect the likelihood of delinquent behavior. In the case of youth, having a job brings about a change in how they spend their time (34, 35). Since employment helps one earn income (29, 36), occupies his/her idle time (37), and modifies his/her routine activities (38), it helps discourage delinquent behavior (39). Also, according to Social Control Theory (31), strong bonds to social institutions likely increase one’s sense of responsibility and restrict him/her from engaging in delinquent behavior. In this sense, employment can be viewed as a social bond, an informal social control that helps alter his/her delinquent trajectory and decreases his/her engagement in delinquent behavior [e.g., Refs. (38, 40, 41)]. Besides, according to Life Course Theory (38, 39, 42), successful completion of life events, such as having completed high school, having been married, and parenthood, can alter one’s life trajectory and subsequent engagement in delinquent behavior. Getting a job is one of the transitional events that imposes an effect on one’s likelihood of engaging in delinquent behavior. All of the above sociological theories provide theoretical foundations for the significant link between employment and decrease in delinquent behavior.

Subsequent scholars also provided support for the association between employment and decrease in delinquent behavior. As stated by some scholars [e.g., Refs. (1, 2, 43)], stable work can be regarded as a form of informal social control that helps one desist from delinquent behavior. Employment encourages social conformity, since delinquents are provided with opportunities to interact with others who are conventional in work situations; this enhances their tendency to conform (38, 44). Further elaborated by Sampson and Laub (38), 141), similar to marriage, employment is “characterized by an extensive set of obligations, expectations, and interdependent social networks,” meaning that employment helps generate new situations where supervision and monitoring are possible. Supported by Ploeger (37), employment discourages delinquent behavior, as it introduces positive work ethics and helps establish bonding to conventional society. For Caspi et al. (45), employment might even promote the chances of experiencing other types of transitions, such as fertility, which may strengthen other forms of informal social bonds that further steer young people away from delinquent behavior (i.e., earning money to support a child’s living). In addition, as stated by Cullen and Travis (46), as well as Phillips and Sandstrom (47), employment helps bring about healthy development of young people, which reduces their delinquent behavior. Employment enhances one’s self-esteem (37, 48); it provides new opportunities for people to receive social support and changes in routine activities, which might promote transformations in identity (38, 48). Summarizing the above illustrations, employment reduces the likelihood of engaging in delinquent behavior by helping one socialize to adaptive norms, increase bonding to social bonds and life transitions, as well as establish a positive identity.

There is a view positing that the relationship between employment and delinquent behavior is a spurious one (e.g., 33, 49, 50) because the relationship is intervened by various preexisting factors. Possible factors are one’s level of self-control (e.g., having low self-control leads to engagement in delinquent behavior due to the urge for enjoying immediate satisfaction at the expense of behavioral consequences) (49) and predisposition for delinquent behavior (50). Some scholars pointed out that one’s perceived meaningfulness of the transitional events, reflective ability, and his/her sense of control over life events affect the change in delinquent trajectories (51, 52). Some scholars even pointed out that employment might increase delinquent behavior [e.g., Refs. (53–55)]. For example, intensive work (e.g., long working hours) likely displaces engagement in schooling and weakens social control (31, 56), which in turn leads to a number of detrimental effects for adolescents including lower level of academic achievement (55, 57), dropping out of school (56, 58), poorer relationships with peers and family, and even poorer health and psychological well-being [e.g., Refs. (55, 59)]. Also, employment may bring about financial resources and increased autonomy (60), which encourage one’s participation in unstructured socializing activities (e.g., partying with friends without the presence of authoritative figures) that serve as opportunities for engaging in delinquent behavior (35). On the other hand, some scholars highlighted the significance of the quality of employment, such as stability (42), salary (4, 61), benefits (62), degree of satisfaction (62, 63), length of working hours [e.g., Refs. (54, 64)], and level of innovation and complexity (65), in the effect on delinquent behavior. Full-time jobs or higher-quality jobs lower the likelihood of recidivism [e.g., Refs. (63, 66–68)]. Explained by Wadsworth (69), (p. 1044), good career development and high-quality jobs encourage one to become more conforming, as they are more “at stake” and had “more to lose.” It enhances one’s engagement and investment to his or her work and brings about high level of satisfaction (50). Also, Agnew (66) pointed out that prestige from occupation reduced delinquent behavior. Besides, according to Agnew (28), jobs that do not create too much stress or psychological burden can reduce delinquent behavior among adolescents, since these situations are more likely to reduce feelings of frustration or anger that might provoke delinquent behavior. Suggested by Mortimer (64), the quality of employment affects youths’ psychosocial well-being; poor quality work likely brings about poor mental health and induces delinquent behavior. In addition, some scholars even stated that good jobs were related to other factors, such as housing and affective relationships, which helped further decrease the likelihood of engaging in delinquent behavior (70, 71). All these notions show that the quality of employment, in terms of career development and sense of satisfaction that it brings, matters when discussing its effect on desistance.

On the contrary, unemployment increases the likelihood of delinquent behavior due to the cumulative disadvantage of long-term distance from the labor market (72). Low-quality work increases the likelihood of delinquent behavior (50). As stated by Crutchfield and Pitchford (43), individuals who have unstable jobs are more likely to engage in delinquent behavior, as they are less likely to be confined by work and have more freedom to linger in places like bars and street corners, which induce the commitment in delinquent acts. Besides, having low aspirations and expectations in work heightens the chance of engaging in delinquent behavior, because there is not much to lose (73).

To summarize, although the relationship between employment and delinquent behavior is inconsistent, there are a number of theoretical notions supporting the idea that employment can help one desist from delinquent behavior. Employment, especially stable employment, brings about a number of positive effects, such as 1) heightening one’s risk involved with offending, increasing his/her pressure to conform; 2) increasing opportunities for monitoring his/her delinquent behavior; 3) decreasing his/her idle time for engaging in delinquent behavior; 4) facilitating his/her experience in other transitional events; 5) experiencing new opportunities for social support and developing new identity; and 6) enhancing his/her self-esteem. Moreover, employment with higher quality and less pressure has a higher effect on desistance.

### Insights from the Above Literature to the Context of Youth in Hidden Situations

Young people in hidden situations, known as hikikomori in Japan (74), are commonly described as having retreated from social participations and connections, including school or work, for at least 6 months (74–76). As suggested by Saito (74), it might not be suitable to attribute youths’ hidden situations to mental illnesses. Although this group of youth is related to NEET (11), they are different from Otaku who are referred to as “notoriously obsessive fans of manga, anime, video games, and other forms of Japanese popular culture” (11, 12). When the phenomenon was uncovered in Hong Kong in 2004, the definitions of these youths were generally similar (77). According to Wong and Ying (12), the hidden situations of these youths are provoked by the inability to fit into the education system or labor market, leading to a lack of social status and becoming Three-lows. Regarding their characteristics, there exist two perspectives of illustrations, namely, the clinical perspective and the nonclinical perspective (77). Scholars supporting the clinical perspective stated that being hidden was associated with psychiatric disorders such as Internet addiction (78), social anxiety (79, 80), autism (81), schizophrenia, and affective disorder (82), while those supporting the nonclinical side held that the culture of society (e.g., rigid expectations on youth regarding their school-to-work transitions, rigid employment practices to which young people find it difficult to conform) [e.g., Refs. (13, 14, 83)] was an important cause of being hidden. Although there are notions that hikikomori or youths in hidden situations lack the ability to maintain a long-term job (84) due to their fear of encountering failures (85), it is also possible that young people actively withdraw because they perceive a mismatch with the employment structure and mainstream standards in society [e.g., Ref. (86)].

On the other hand, in Hong Kong and Japan, these youths have been reported as having committed delinquent behavior, such as killing (87) and violence (88). Based on the relationship between employment and delinquent behavior as described previously [e.g., Refs. (63, 66–68)], it implies that youth in hidden situations have a high tendency to engage in delinquent behavior due to their unemployment. However, researchers found that, from previous practice experience with youths in hidden situations, some of these youths engage in home-based work; also, they have low propensity to engage in delinquent behavior. This suggests that these youth are not homogenously jobless and delinquent-prone as portrayed in society. In fact, as society changes, home-based, Internet-based work and freelance work have become increasingly prevalent in contemporary society (19–21). This means that jobs nowadays are no longer restricted to traditional, typical forms of work arrangement (e.g., requiring a workplace outside home) (24, 4). It is plausible for young people in hidden situations to work at home with their computers during prolonged seclusion. Owing to the lack of previous studies that investigate these youths’ employment situations and the subsequent effects on their delinquent behavior, this study aims to fill this research gap. It is expected that this study can 1) de-stigmatize these youth as “lacking social status” and “being unable to work” and 2) refine the meaning of employment so as to respond to the societal changes nowadays.

## Method

### Participants

There were 540 participants in this study, who were recruited through purposive sampling. This sampling method is useful for locating “unusual” extreme or deviant cases (89, 182), such as young people in hidden situations who only appear online but are *invisible* in social situations. Eligible participants should be residents of Hong Kong, be within the age range of 12–30, have retreated from society for at least 6 months, and have received no psychiatric diagnosis or treatment. The criteria for youth in Hong Kong in the context of social service provision is 12–29 years old. Hence, the participants in the study were termed as “youths” or “young people” in hidden situations.

Of the total participants, 64.1% (*N* = 346) were male, while the rest (35.9%, *N* = 194) were female. More than half of them (61.7%, *N* = 333) were 21 or older. Participants in this study had withdrawn for 1 to 8 years; 51.7% (*N* = 279) had been hidden for 1–2 years, while 48.4% (*N* = 261) had been hidden for longer than 2 years. For their level of hidden situation, 15.9% (*N* = 86) of participants were identified as being in a level 1 hidden situation (i.e., the lowest level); 73.0% (*N* = 394) were at levels 2 to 4, and 11.1% (*N* = 60) were at level 5 (i.e., the highest level). Regarding their education level, 53.1% of them reported to have achieved senior secondary qualification (*N* = 287), while 29.4% (*N* = 159) and 17.4% (*N* = 94) of them had achieved the education qualification of college/university or above and junior secondary, respectively. With respect to their family income, only 37.2% (*N* = 201) received a family income of HK$20,000 or less; more than half of them (62.7%, *N* = 339) had a family income of HK$20,000 or more (US$1 = HK$7.8). For their employment status, although most of them (83.5%, *N* = 451) reported to have no employment, there were some (15.1%, *N* = 89) who had work, with most of them (87.6%, *N* = 78) having Internet-based jobs and a few of them (10.1%, *N* = 9) being self-employed. This reflects that young people are not necessarily lacking in social status (11) or refusing social participations (76), but they have working ability and employability. For their criminal records, all of the participants had no history of committing delinquent behavior.

### Data Collection and Procedure

Participants were own cases from a local service center established by the researcher, who were mainly located via Internet platforms (e.g., forums and online gaming platforms). Due to prior understanding of the participants, researchers were able to understand their employment situations and delinquent behavior.

Informed consent from participants was achieved by directly giving consent forms to those who had reached 18 years of age and giving the forms to their parents if they had not reached 18. Researchers also met them face-to-face, to explain the aim, topic, and process of the study, and assess their eligibility for participating in the study. Once all of the aforementioned procedures were completed, the questionnaire was administered to them.

On the other hand, 56 participants participated in the qualitative study. The interviews were conducted in a face-to-face manner.

### Measurement Scales

#### Employment

In this study, the transitional event of employment was examined to see if it affected participants’ involvement in delinquent behavior in life. This piece of information was collected from participants by asking them to self-report whether they had a job in the questionnaire.

In this study, participants who had full-time work and earned a living from their work were considered as employed. Among the youths in hidden situations, Internet-based work and freelance jobs, which are non-typical work arrangements, are common.

#### Period and Level of Hidden Situation

Period of hidden situation refers to the duration of being hidden. This piece of information was obtained from a question about how long they had retreated from society in the questionnaire. For the level of hidden situation, according to Oiwa (90), there are five levels of hidden situation, with item 1 representing the lowest level of hidden situation (i.e., “In the past six months, I have not gone outside”) and item 5 representing the highest level (i.e., “In the past six months, I have not talked to anybody”). One’s level of hidden situation indicates the amount of social relationships and social support that one possesses during hidden situation. Prolonged seclusion does not necessarily mean that the youths will not maintain online connections or participate in online work.

#### Delinquent Behavior

The Youth Deviant Behavior Scale of Yang (91) was used to assess participants’ engagement in any type of delinquent behavior in the past year. This scale was used owing to its suitability for Asian contexts like Hong Kong. This scale consists of three subscales, including externalizing deviant behavior (30 items, e.g., fighting and drug-taking), internalizing deviant behavior (18 items, e.g., engaging self-harm behavior and having suicidal attempts), and difficulty in academic adaptation (12 items, e.g., sleeping in class). Considering the applicability of the scale, only subscales of externalizing deviant behavior and internalizing deviant behavior were used, and only items relevant to delinquent behavior were included in the analysis. All items adopt a five-point Likert scale (1 = never; 2 = 1 to 3 times; 3 = 4 to 6 times; 4 = 7 to 9 times; 5 = more than 10 times).

Delinquent behavior, by definition, is law-breaking behavior (26). Not all self-reported delinquent behaviors cause official arrests and are included as criminal records (92). In this study, participants’ self-reported delinquent behavior was adopted because the study aims at investigating the social determinants of delinquent behavior among youth in hidden situations (i.e., employment status). The details of the participants’ self-reported delinquent behavior are presented in **Table 1**.

**Table 1:** Sample characteristics (*N* = 588).

Variables	%	*N*
Gender		
Male	64.1	346
Female	35.9	194
Age		
16–20	38.3	207
21–25	60.0	324
26–27	1.7	9
Education		
Junior secondary (Years 7–9)	17.4	94
Senior secondary (Years 10–11)	39.6	214
Matriculation (Year 13)	13.5	73
College/university or above	29.4	159
Family income (USD$1 = HK$7.8)		
Below HK$10,000	16.1	87
HK$10,001–20,000	21.1	114
HK$20,001–30,000	23.1	125
HK$30,001 or above	39.6	214
Earning ability		
Yes	49.3	266
No	50.7	274
Criminal records		
Yes	0.0	0
No	100.0	540
Period of hidden situation		
1 year	27.8	150
2 years	23.9	129
3 years	8.3	45
4 years	15.4	83
5 years	11.7	63
6 years	5.6	30
7 years	5.0	27
8 years	2.4	13
Level of hidden situation		
1	15.9	86
2	14.1	76
3	30.2	163
4	28.7	155
5	11.1	60
Holding full-time employment		
Yes	16.5	89
No	83.5	451
Types of full-time employment (*N* = 89)		
Internet-based work	87.6	78
Self-employed	10.1	9
Unknown	2.2	2
Types of delinquent behavior committed		
Theft	1.5	8
Drug taking	5.9	32
Fraud	0.9	5
Online fraud/theft	4.1	22
Hacking	10.9	59
Spreading virus online deliberately	17.8	96
Reading pornography	11.1	60
Online gambling	7.8	42
Others	3.9	21

Reading pornography includes downloading and distributing pornographic materials.

### Quantitative Analysis

SPSS for Windows 19.0 was the program used for conducting the statistical analyses, in which *p* < .05 was the significance level of the study. Analyses included the following: 1) cross-tabulation analysis was conducted to see the distribution of participants in employment in different periods and levels of hidden situation; 2) Spearman’s rank correlation was performed to investigate whether employment was associated with participants’ period and level of hidden situation, as well as their engagement in delinquent behavior; and 3) mediation analysis was undertaken to examine whether participants’ employment contributes to the link between hidden behavior and delinquent behavior, so as to shed light on the importance of employment in affecting their engagement in delinquent behavior.

### Qualitative Analysis

Other than the quantitative analyses, qualitative verbatim accounts of the participants were analyzed to examine how their jobs influenced their involvement in delinquent behavior. To conduct the qualitative analysis, the recordings of the interview were first transcribed, and then the verbatim data of the participants were summarized in terms of the reasons for not engaging in delinquent behavior.

## Results

### Employment and Hidden Situation

**Figure 1:**
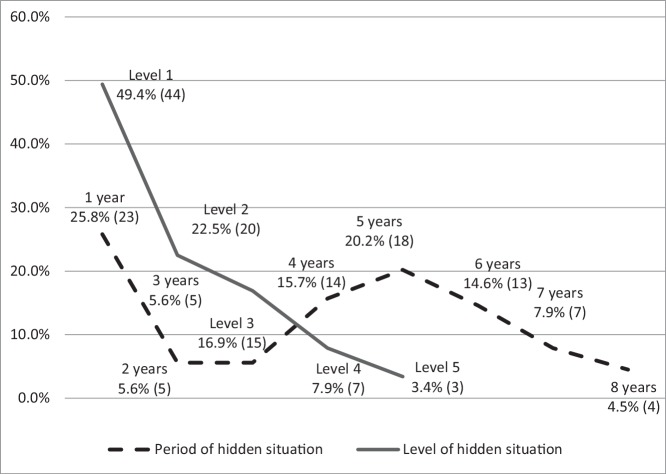
Participants’ employment status in different periods of hidden situation (in terms of year) and levels of hidden situation (*N* = 89).

**Figure 1** shows the distribution of employment of participants in the different periods and levels of hidden situation. With respect to the period of hidden situation, about one-fourth of participants (25.8%) who had a job had 1 year of hidden experience; although only a small number of participants (11.2%) with 2 to 3 years of hidden experience had a job, over 40% (47.2%) of the participants who had a job had been hidden for 5 years or more. This reflects that although young people retreat from social participation when they start to become hidden, they will have jobs again when they stay in a prolonged hidden situation. On the other hand, regarding the level of hidden situation, results showed that as the level of hidden situation increased, the number of participants engaging in employment decreased. This reflects that fewer young people who are in a deeper level of hidden situation have jobs.

The above results suggest that young people in hidden situations engage in employment. In the following, correlation analysis was performed to investigate whether participants’ employment is related to their engagement in delinquent behavior.

### Employment and Delinquent Behavior

Employment was negatively correlated to delinquent behavior (*r* = −.41). This shows that having a job is related to a decrease in delinquent behavior. Additionally, period of hidden situation was negatively correlated to delinquent behavior (*r* = −.38) but positively correlated to employment (*r* = .17), while level of hidden situation was positively correlated to delinquent behavior (*r* = .74) but negatively correlated to employment (*r* = −.39). These findings reflect that as young people progress to a hidden situation, they are more likely to hold jobs and their involvement in delinquent behavior may decrease, while as the level of hidden situation increases, young people are less likely to hold jobs and their involvement in delinquent behavior may increase.

The results above show that youths in hidden situations can attain jobs, and they show lower engagement in delinquent behavior. At the same time, for those having a higher level of hidden situation, fewer of them get into employment and they exhibit more involvement in delinquent behavior. In order to further explore the effect of employment on the tendency to engage in delinquent behavior in terms of period and level of hidden situation, mediation analysis was conducted in the following section.

### The Role of Employment in the Relationship Between Hidden Behavior and Delinquent Behavior

In this analysis, the proposed mediation path was “hidden behavior → employment → delinquent behavior.” Both period and level of hidden situation were tested. To assess the mediation properties of outcome expectancies, *PROCESS Macro for SPSS* (93) was used. The proportion of indirect effect on total effect was derived by expressing the ratio of indirect to total effect of X on Y as performed by the Macro (93). Also, to test the significance of the proposed mediation paths, Sobel tests were undertaken (94).

**Figure 2:**
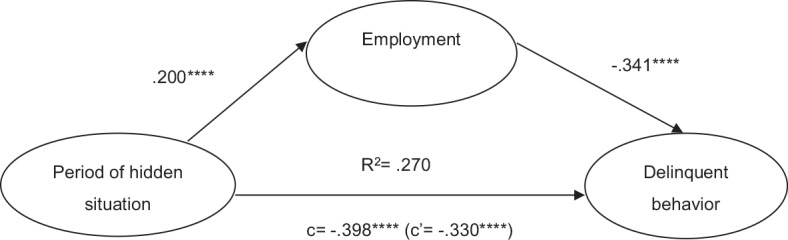
Mediation model of period of hidden situation, employment, and delinquent behavior (Path 1). ****p = .0000.

**Figure 3:**
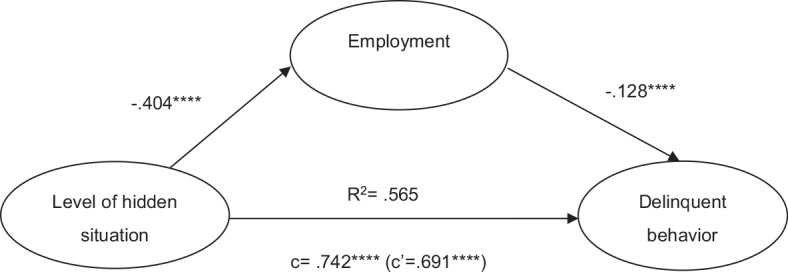
Mediation model of level of hidden situation, employment, and delinquent behavior (Path 2). ****p = .0000.

The indirect effect in each mediation path was found to be significant. Results are shown in **Table 2** and **Figures 2** and **3**.

**Table 2:** Standardized estimates of direct and indirect effects on delinquent behavior and mediator (Paths 1 and 2).

	Effect	% Explained of total effect	Sobel Test
Hp→Em→Delin be (total effect)	−.398		
Hp→Em→Delin be (indirect effect)	−.068	17%	−6.833****
Hl→Em→Delin be (total effect)	.742		
Hl→Em→Delin be (indirect effect)	.051	7%	3.186**

Hp, period of hidden situation; Hl, level of hidden situation; Em, employment; Delin be, delinquent behavior. **p < .01. ****p = .0000.

As shown in **Figure 2**, period of hidden situation negatively predicted delinquent behavior (⹌β = −.40). With employment having existed as the mediator, the effect of period of hidden situation on delinquent behavior diminished (⹌β = −.33). In the relationship between period of hidden situation and delinquent behavior, employment was a significant mediator, accounting for 17% of the relationship. Period of hidden situation displayed a positive expectancy of employment (⹌β = .20), while employment displayed a negative expectancy of delinquent behavior (⹌β = −.34). This shows that as participants withdraw for a longer period, their engagement in delinquent behavior decreases; employment contributes to lowering the engagement in delinquent behavior among these young people.

As shown in **Figure 3**, level of hidden situation showed a positive expectancy of delinquent behavior (⹌β = .74). With employment included as the mediator, the impact of level of hidden situation on delinquent behavior was attenuated (⹌β = .69). Employment was a significant mediator in the relationship between level of hidden situation and delinquent behavior, explaining 7% of the relationship. Level of hidden situation negatively expected employment (⹌β = −.40), and employment negatively predicted delinquent behavior (⹌β = −.13). This suggests that participants at a higher level of hidden sit­uation show a higher likelihood of involving in delinquent behavior; this is because fewer of these young people become employed.

The results of the mediation analyses suggest that although fewer young people at a higher level of hidden situation have jobs, those who stay in hidden situations hold employment. Work prevents them from engaging in delinquent behavior. Besides, comparing the two mediation analyses, the mediating effect of employment is slightly more prominent in the relationship between period of hidden situation and delinquent behavior than that between level of hidden situation and delinquent behavior. This means that the prohibiting effect of employment on delinquent behavior is slightly larger among young people who have a lower level of hidden situation.

The following qualitative verbatim accounts of participants illustrated different reasons of not engaging in delinquent behavior when holding employment:

#### Not Engaging in Delinquent Behavior due to the Structured Environment of Work Where Supervision Is Allowed

No (not engaging in delinquent behavior anymore). Although I don’t need to go to work, drawing graphics is time-consuming and I need to pay much attention to this. If I take drugs, I will not be able to concentrate on my work, and will be scolded. (Youth b)

…now I work for 14 hours every day (as a shop assistant in an online store). I won’t do those meaningless things (taking drugs) again. In the past, I did this (drug-taking) to kill my time. Now I need not do this anymore. (Youth c)

I love my job (working as a translator)…I don’t want to do anything which makes me lose my job. (Youth j)

As stated by Youth b and Youth c, jobs reduced their idle time to commit delinquent acts. At the same time, the verbatim accounts of Youth b reflected that work situations had a supervising effect on their behavior, which prohibited them from becoming involved in delinquent behavior, such as drug-taking. Delinquent behavior could affect work performance. Also, Youth j reported that having a job increased his cost for engaging in delinquent behavior, as he feared that being delinquent would make him lose his job.

#### Not Engaging in Delinquent Behavior due to the Establishment of a New Self and New Life Resulted From Employment

Now I have different social circles. My friends become different. So I no longer engage in hacking activities and set cheating programmes… (Youth a)

…in the past, I fooled around in BBS (Bulletin Board System) with my peers, criticizing and teasing other people as a pastime. At that time, I thought that I had status on the Internet. However, when I have a job, I think that BBS becomes no longer important. I think that my value can be found elsewhere. (Youth e)

In the past, I used the same skill (stealing passwords from other players’ accounts in online games) to find money, but it’s illegal; now I use the same skills and experience in my work (as a game manager), and it’s legal. I feel great! (Youth g)

I love my job (working as a translator), and I can enjoy my beloved lifestyle while being able to support my life… (Youth j)

As stated by Youth a, the job created opportunities for him to build new social networks, which drove him away from delinquent behavior. As expressed by Youth e, jobs brought them a new identity, value, and meaning of life. As expressed by Youth g and Youth k, having legal jobs increased their self-esteem (Youth g). Besides, the verbatim accounts of Youth j reflected that he enjoyed his work, as it brought him a high quality of life. This implies that work of high quality helps prevent one from becoming helpful in delinquent behavior.

#### Other Reasons for Not Engaging in Delinquent Behavior due to Employment

Of course not (not engaging in delinquent behavior anymore). I do this (search for bugs) every day when I’m at work. No kidding! (He works as a programmer who is responsible for finding bugs in online games). (Youth d)

In the past, I didn’t earn money by myself, so I didn’t know how difficult money-making is … Now I earn $250 a day, which means that I can only get half an ounce of ketamine … so expensive! So I don’t take ketamine anymore now. (Youth i)

Now I’m more concerned about other people’s feelings. I’ve learnt to take other people’s perspectives before saying and doing anything. So I think that I was wrong (to uncover and bully other people on the Internet) before. (He works as a peer counselor) (Youth l)

On the other hand, participants showed other reasons for not engaging in delinquent behavior. For Youth d, employment offered him a legitimatized way to engage in bug-finding. Since his interests, potentials, and needs had already been fulfilled in his job, his drive to engage in delinquent behavior was weakened. For Youth i and Youth l, they gave up delinquent behavior including drug-taking (Youth i) and cyber bullying (Youth l) as they understood the importance of saving money (Youth i) and perspective-taking (Youth l), respectively. They thought that engaging in delinquent behavior wasted money (Youth i) and brought harm to others (Youth l).

In summary, all of the above verbatim accounts of participants show that young people in hidden situations have jobs, such as graphic design (Youth b), working as an online shop assistant (Youth c), programming (Youth d), webpage designing (Youth f), working as a game manager (Youth g), making cosplay costumes (Youth h), translation (Youth j), working as a sensory operator on the gaming platform (Youth k), and working as a peer counselor (Youth l). Participants have various reasons for ceasing delinquent behavior due to employment. In general, the reasons show coherence to the literature review, including increased sense of satisfaction brought about by the job, being more “at stake” (69, 1,044), and establishment of a new life and identity due to employment. Although the money earned from jobs might create opportunities for engaging in delinquent behavior [e.g., Ref. (60)], it does not apply in the case of the youth participants. It is the nature, quality, and meaningfulness of their jobs, as well as their outweighing of benefits and drawbacks of being delinquent, that effectively prohibit them from engaging in delinquent behavior.

## Discussion and Conclusion

Results showed that 15.1% of youths in hidden situations had jobs. They mainly worked in an Internet-based, home-based manner, with some of them being self-employed or working as freelancers, and they were able to make money from their jobs. This suggests that these youths’ being jobless is a prejudice; they are not necessarily those who lack social status as described in previous literature [e.g., Refs. (11, 12, 76)] but can engage in work during prolonged seclusion as their preferred lifestyle and use it to support their lives. Also, the relationship between employment and delinquent behavior was significant in the context of youths in hidden situations. Employment serves as a form of informal social control (38) or “meaningful social attachment” (95, 381) to reduce the likelihood of being involved in delinquent behavior by creating opportunities for supervising behavior, increasing the risk for offending, decreasing the idle time for offending, and allowing new opportunities to build new social networks. Also, employment helps youths in hidden situations to transform, develop a new identity, develop a new set of values, and enhance their self-esteem (37, 38, 48). Additionally, it is noted that the quality of jobs plays a part in these youth’s desistance in delinquent acts (28, 50). As stated in previous research, the importance of employment in reducing delinquent behavior is the positive experience that employment brings (e.g., perceiving a job as good and fulfilling, and enhancing self-worth), not just the nature of employment (e.g., full-time or part-time) (64, 96). In the case of the youths, it appears that not only the structure and stability of their jobs (e.g., working hours) are significant—when they love their jobs and think that their jobs help them pursue their preferred lifestyle, their employment can serve as a form of informal social control that effectively displaces the delinquent lives they used to have. All these suggest that the relationship between employment and delinquent behavior as stated in the existing literature could be applied in the context of youth in hidden situation. The work engaged by the youths in hidden situations displayed the same impact on reducing delinquent behavior as stated in traditional theories.

The results about the youths’ employment situations provoke reflections upon the concept of employment in Hong Kong. According to the Census and Statistics Department (23), “employed population” refers to “persons aged 15 and over who have been at work for pay or profit during the 7 days before enumeration or who have had formal job attachment”; although there are no clear definitions of “formal job attachment,” employment is commonly recognized as “working outside” [e.g., Ref. (24, 4)]. Also, despite the fact that the Hong Kong Standard Industrial Classification Version 2.0 (HSIC V2.0) has covered a variety of categories of industries (20 broad categories) including primary (e.g., “Agriculture, forestry and fishing”), secondary (e.g., “Manufacturing” and “Construction”), and tertiary industries (e.g., “Information and communications” and different service industries) (97, 12), the delineation of the categories is mostly based on the structure of the local economy (23). From the above definitions of employment, it is noticed that although various types of industries are recognized, the mode of work appears to be exclusive of home-based work. A probable result of this definition will be that some jobs, such as home-based work and Internet-based work, will not receive social recognition and these people will still be perceived as jobless or having low motivation to work even though they have contributed their labor to earn a living. In fact, alongside technological advancement, it is well known that Internet-and home-based work is increasingly popular among people in contemporary society (98); owing to the lifestyle of youths in hidden situations, these youths even play a part in pioneering this trend. Hence, in response to societal changes, the understanding of employment should change accordingly and the stereotypical understanding of employment among the general public should be overturned.

To conclude, the results of this study show that young people in hidden situations, though not all of them, have work. This helps de-stigmatize them as a group of jobless youth who are socially isolated and have a fear of entering the labor market. Although, from a clinical perspective, prolonged hidden situation would lead to negative consequences including mental issues (e.g., depression and social anxiety), social isolation, and fear of engaging in interpersonal relationships [e.g., Refs. (99, 100)], it is pointed out that as long as these youths can find out the interests and meaningfulness of work, they will be motivated to get a job again (101), which in turn buffers them from engaging in delinquent behavior. According to previous studies on youths in hidden situations (77, 102), being able to pursue the self-preferred interests during prolonged hidden situation brings about an enhanced sense of well-being and quality of life. Hence, the dominant judgments of these youth might no longer apply. Instead, it is necessary to view the young people in hidden situations and even the concept of employment from a brand-new angle. To address these implications, firstly, during practice, instead of encouraging young people in hidden situations to “re-engage into the mainstream society,” practitioners can try to adopt a youth-oriented perspective to appreciate their choice of work life and the subsequent positive effects; meanwhile, practitioners can undergo parental education, to educate the parents of these youth about how they can support their children’s growth and development through allowing them to pursue the work they prefer, rather than forcing their children to step out of home and enter the mainstream labor market, which may not suit these young people’s needs. Through this kind of intervention, it not only helps advocate a change in the public concept about youths in hidden situations but also helps nurture positive growth and development of the youth as it acknowledges their uniqueness and individuality. Most importantly, owing to the positive values of home-based or Internet-based work engaged by youths in hidden situations, it is advocated that the definition of “employment” must be revised and enriched, with the meaning of formal work being extended to home-based work, Internet-based work, and freelance jobs, so as to respond to the societal changes and embrace the diversity in the concept of employment in society. The modification of the definition of employment will benefit not only youths in hidden situations but also other groups of youths who had jobs of similar nature.

## Limitations and Future Studies

The sampling method used in this study likely constitutes the limitation of this study. Since the participants were mainly recruited through the Internet, the sample might probably not be able to include young people in hidden situations who do not use the Internet. As such, the representability of the sample might be hindered. Hence, in the future, similar studies can be performed on participants who are recruited by other types of sources to see whether similar results can be derived.

Also, it is mentioned that the relationship between employment and delinquent behavior is affected by other factors, such as level of self-control and delinquent propensity [e.g., Refs. (49, 50)]; also, it is even pointed out that prior identity transformation (e.g., perceiving the delinquent career as costly rather than beneficial) must exist before employment serves as an effective social bond to buffer against delinquent behavior (103–105). To see if this also applies in the case of youths in hidden situations, in future studies these factors can be incorporated into the analysis, so as to further enrich the research of youths in hidden situations in terms of the relationship between employment and delinquent behavior (e.g., the intervening effect of identity change and employment in these youths’ desistance in delinquent behavior).

## Ethics Statement

This study was carried out in accordance with the recommendations of the “Research and Ethics Committee, City University of Hong Kong,” with written informed consent from all subjects. All subjects gave written informed consent in accordance with the Declaration of Helsinki. The protocol was approved by the “Research and Ethics Committee, City University of Hong Kong.”

## Author Contributions

The author confirms being the sole contributor of this work and has approved it for publication.

## Conflict of Interest Statement

The author declares that the research was conducted in the absence of any commercial or financial relationships that could be construed as a potential conflict of interest.

## References

[1] Irwin J The felon. Englewood Cliffs, NJ: Prentice-Hall (1970).

[2] Shover N Great pretenders: pursuits and careers of persistent thieves. Boulder, CO: Westview Press (1996).

[3] Veysey BM Martinez DJ Christian J Getting out: a summary of qualitative research on desistance across the life course. In: Gibson CL Krohn MD, editors. Handbook of life-course criminology: emerging trends and directions for future research. New York: Springer (2013). p. 233–60. 10.1007/978-1-4614-5113-6_14

[4] Grogger J Market wages and youth crime. J Labor Econ (1998) 16(4):756–91. 10.1086/209905

[5] Junger M Marshall IH The interethnic generalizability of social control theory: an empirical test. J Res Crime Delinq (1997) 34(1):79–112. 10.1177/0022427897034001005

[6] Berk R Lenihan KJ Rossi PH Crime and poverty: some experimental evidence from ex-offenders. Am Sociol Rev (1980) 45:766–86. 10.2307/2094894

[7] Piliavin I Gartner R The impact of supported work on ex-offenders. Madison, WI: Institute for Research on Poverty and Mathematical Policy Research (1981).

[8] Kruttschnitt C Uggen C Shelton K Predictors of desistance among sex offenders: the interaction of formal and informal social controls. Justice Q (2000) 17:61–87. 10.1080/07418820000094481

[9] Needels K Go directly to jail and do not collect? A long-term study of recidivism, employment and earning patterns among prison releases. J Res Crime Delinq (1996) 33:471–96. 10.1177/0022427896033004005

[10] Chan GHY Lo TW Hidden youth in Hong Kong, negative emotions, and deviant behavior. Issues on Juvenile Crimes and Delinquency (2014a) 3:43.

[11] Tsutsui WM Nerd nation Otaku and youth subcultures in contemporary Japan. Educ Asia (2008) 13(3):12–8.

[12] Wong V Ying W Young people and social withdrawal: a social exclusion perspective. Hong Kong J Soc Work (2006) 40(1/2):61–91. 10.1142/S0219246206000064

[13] Dziesinski MJ Hikikomori: investigations into the phenomenon of acute social withdrawal in contemporary Japan (2003). Retrieved 14 May 2014 from http://towakudai.blogs.com/Hikikomori.Research.Survey.pdf.

[14] Zielenziger M Young Japanese prefer “parasite single” life to “wedding poverty”. In: Knight Ridder/Tribune News Service. San Jose, California: Knight Ridder (2002). December 18. Retrieved 14 May 2014 from http://www.highbeam.com/doc/1G1-95624312.html.

[15] Japanese Ministry of Education, Culture, Sports, Science and Technology Kongo-no futōkō-e no taiō-no arikata-ni tsuite (Hōkoku). Tokyo, Japan: Ministry of Education, Culture, Sports, Science and Technology (2003). Retrieved 14 May 2014 from http://www.mext.go.jp/b_menu/public/2003/03041108.htm/.

[16] Teo AR Fetters MD Stufflebam K Tateno M Balhara Y Choi TY Identification of the Hikikomori syndrome of social withdrawal: psychosocial features and treatment preferences in four countries. Int J Soc Psychiatry (2015) 61(1):64–72. 10.1177/0020764014535758 24869848 PMC5573567

[17] Yong R Exploring Hikikomori – a mixed methods qualitative approach. Int J Behav Med (2010) 17:81–2. 10.5353/th_b4171214

[18] Wong V Young people in social withdrawal - an extreme form of social exclusion? Policy agenda and organizational practices. Paper presented at EASP 5th Conference, Welfare Reform in East Asia, EASP Taipei, Taiwan: National Taiwan University (2008).

[19] European Foundation for the Improvement of Living and Working Conditions Non-standard forms of employment: recent trends and future prospects. Dublin: Author (2017).

[20] Legislative Council Panel on Manpower Policy Study on standard working hours. LC Paper No. CB(2)341/12-13(07) (2012). Retrieved 3 August 2018 from https://www.legco.gov.hk/yr12-13/english/panels/mp/papers/mp1218cb2-341-7-e.pdf.

[21] Maxwell G Rankine L Bell S Mac Vicar A The incidence and impact of flexible working arrangements in smaller businesses. Empl Relat (2007) 29(2):138–61. 10.1108/01425450710719987

[22] Chan GHY The effect of life-course transitions on delinquent behavior among youth in social withdrawal situation. Deviant Behav (2015) 36(12):935–55. 10.1080/01639625.2014.977181

[23] Census and Statistics Department Labor. Hong Kong: Census and Statistics Department (2016). Retrieved 22 August 2017 from http://www.censtatd.gov.hk/hkstat/sub/sc30.jsp.

[24] Business, Economic and Public Policy Research Center, Hong Kong Shue Yan University (BEPP), Competitiveness of Youth in Hong Kong (1^st^ Stage). Hong Kong: Business, Economic and Public Policy Research Center, Hong Kong Shue Yan University [BEPP] (2013). Retrieved 14 May 2014 from http://www.coy.gov.hk/filemanager/template/common/images/research/competitiveness_of_youth_in_hk_201304.pdf.

[25] Meier R Cain CM Deviance, normative definitions of. In: Ritzer G, editor. The Blackwell encyclopedia of sociology. 2nd ed Hoboken, NJ: John Wiley & Sons, Ltd (2015). 10.1002/9781405165518.wbeosd051.pub2

[26] Yang GS School factors affecting problematic behavior among junior high school students. In: Dissertation Series of Youth Problem in Social Change Forum: Institute of Ethnology Academia Sinica., vol. 24 Taipei, Taiwan: Institute of Ethnology, Academia Sinica (1978). p. 33–55.

[27] Humphrey JA Schmalleger F Deviant behavior. Sudbury, MA: Jones & Bartlett Learning (2012).

[28] Agnew R Foundation for a general strain theory of crime and delinquency. Criminology (1992) 30:47–87. 10.1111/j.1745-9125.1992.tb01093.x

[29] Agnew R Building on the foundation of general strain theory: specifying the types of strain most likely to lead to delinquency. J Res Crime Delinq (2001) 38:319–61. 10.1177/0022427801038004001

[30] Cohen LE Felson M Social change and crime rate trends: a routine activity approach. Am Sociol Rev (1979) 44:588–608. 10.2307/2094589

[31] Hirschi T Causes of delinquency. Berkeley, CA: University of California Press (1969).

[32] Merton RK Social structure and anomie. Am Sociol Rev (1938) 3:672–82. 10.2307/2084686

[33] Paternoster R Bushway S Brame R Apel R The effect of teenage employment on delinquency and problem behaviors. Soc Forces (2003) 82(1):297–335. 10.1353/sof.2003.0104

[34] Osgood DW Having the time of their lives: all work and no play? In: Booth A Crouter AC Shanahan MJ, editors. Transitions to adulthood in a changing economy: no work, no family, no future? Westport, CT: Praeger (1999). p. 176–86.

[35] Osgood DW Wilson JK O’Malley PM Bachman JGA Johnston LD Routine activities and individual deviant behavior. Am Sociol Rev (1996) 61:635–55. 10.2307/2096397

[36] Agnew R Pressured into crime: an overview of general strain theory. Los Angeles, CA: Roxbury (2006).

[37] Ploeger M Youth employment and delinquency: reconsidering a problematic relationship. Criminology (1997) 35:659–75. 10.1111/j.1745-9125.1997.tb01234.x

[38] Sampson RJ Laub JH Crime in the making: pathways and turning points through life. Harvard University Press: Cambridge, MA (1993).

[39] Laub JH Sampson RJ Shared beginnings, divergent lives; delinquent boys to age 70. Cambridge, MA: Harvard University Press (2003).

[40] Barry M Youth transitions: from offending to desistance. J Youth Stud (2010) 13(1):121–36. 10.1080/13676260903233712

[41] Sampson RJ Laub JH Urban poverty and the family context of delinquency: a new look at structure and process in a classic study. Child Dev (1994) 65:523–40. 10.2307/1131400 8013238

[42] Sampson RJ Laub JH Crime and deviance over the life course: the salience of adult social bonds. Am Sociol Rev (1990) 55(5):609–27. 10.2307/2095859

[43] Crutchfield RD Pitchford SR Work and crime: the effects of labor stratification. Soc Forces (1997) 76:93–118. 10.1093/sf/76.1.93

[44] Warr M Life course transitions and desistance from crime. Criminology (1998) 36:83–216. 10.1111/j.1745-9125.1998.tb01246.x

[45] Caspi A Elder GH Jr. Herbener ES Childhood personality and the prediction of life-course patterns. In: Robins LN Rutter M, editors. Straight and devious pathways from childhood to adult life. Cambridge, UK: Cambridge University Press (1990). p. 13–35.

[46] Cullen FT Travis LF III Work as an avenue of prison reform. N Engl J Crim Civ Confin (1984) 10:45–64.

[47] Phillips S Sandstrom KL Parental attitudes toward youth work. Youth Soc (1990) 22:160–83. 10.1177/0044118X90022002003

[48] Farrall S Rethinking what works with offenders. Cullompton, UK: Willan Publishing (2002).

[49] Gottfredson MR Hirshi T A general theory of crime. Stanford, CA: Stanford University Press (1990).

[50] Lustig K Liem JH Quality of employment and delinquency during the adolescent to young adult transition. New School Psychol Bull (2010) 8(1):4–14.

[51] Liebregts N van der Pol P de Graaf R van Laar M van den Brink W Korf DJ Persistence and desistance in heavy cannabis use: the role of identity, agency, and life events. J Youth Stud (2015) 18(5):617–33. 10.1080/13676261.2014.992320

[52] Lloyd CD Serin RC Agency and outcome expectancies for crime desistance: measuring offenders’ personal beliefs about change. Psychol Crime Law (2012) 18(6):543–65. 10.1080/1068316X.2010.511221

[53] Bachman JG Bare DE Frankie EI Correlates of employment among high school seniors. Ann Arbor, MI: Institute for Social Research (1986).

[54] Bachman JG Schulenberg J How part-time work intensity relates to drug use, problem behavior, time use, and satisfaction among high school seniors: are these consequences or merely correlates? Dev Psychol (1993) 29:220–35. 10.1037/0012-1649.29.2.220

[55] Mortimer JT Finch MD The development of self-esteem in the early work career. Work Occup (1986) 13:217–39. 10.1177/0730888486013002003

[56] Marsh HW Employment during high school: character building or subversion of academic goals? Sociol Educ (1991) 64:172–89. 10.2307/2112850

[57] Carr RV Wright JD Brody CJ Effects of high school work experience a decade later: evidence from the National Longitudinal Survey of Youth. Sociol Educ (1996) 69:66–81. 10.2307/2112724

[58] Chaplin DD Hannaway J High school employment: meaningful connections for at-risk youth. Washington, DC: Urban Institute (1996).

[59] Greenberger E Steinberg LD Vaux A McAuliffe S Adolescents who work: effects of part-time employment on family and peer relations’. J Youth Adolesc (1980) 9:189–202. 10.1007/BF02088464 24318075

[60] Longest KC Shanahan MJ Adolescent work intensity and substance use: the mediational and moderational roles of parenting. J Marriage Fam (2007) 69:703–20. 10.1111/j.1741-3737.2007.00401.x

[61] Gould ED Weinberg BA Mustard DB Crime rates and local labor market opportunities in the United States: 1979–1997. Rev Economics and Statistics (2002) 84(1):45–61. 10.1162/003465302317331919

[62] Wadsworth T The meaning of work: conceptualizing the deterrent effect of employment on crime among young adults. Sociol Perspect (2006) 49(3):343–68. 10.1525/sop.2006.49.3.343

[63] Uggen C Ex-offenders and the conformist alternative: a job quality model of work and crime. Soc Probl (1999) 46(1):127–51. 10.2307/3097165

[64] Mortimer J Working and growing up in America. Cambridge, MA: Harvard University Press (2003).

[65] Mortimer JT Finch MD Shanahan M Ryu S Work experience, mental health, and behavioral adjustment in adolescence. J Res Adolesc (1992) 2(1):25–57. 10.1207/s15327795jra0201_2

[66] Agnew R Work and delinquency among juveniles attending school. J Crim Justice (1986) 9:19–41. 10.1080/0735648X.1986.9721321

[67] Uggen C Work as a turning point in the life course of criminals: a duration model of age, employment, and recidivism. Am Sociol Rev (2000) 67:529–46. 10.2307/2657381

[68] Wright JP Cullen FT Employment, peers, and life-course transitions. Justice Q (2004) 21(1):183–205. 10.1080/07418820400095781

[69] Wadsworth T Labor markets, delinquency, and social control theory: an empirical assessment of the mediating process. Soc Forces (2000) 78:1041–66. 10.1093/sf/78.3.1041

[70] Graffam J Shinkfield AJ Lavelle B McPherson W Variables affecting successful reintegration as perceived by offenders and professionals. J Offender Rehabil (2005) 40:147–71. 10.1300/J076v40n01_08

[71] Visher C Travis J Life on the outside: returning home after incarceration. Prison J (2011) 91:102–19. 10.1177/0032885511415228

[72] Sampson RJ Laub JH A life-course theory of cumulative disadvantage and the stability of delinquency. In: Thornberry TP, editor. Developmental theories of crime and delinquency. New Brunswick, NJ: Transaction (1997). p. 1–29.

[73] Agnew R Reflection on “a revised strain theory of delinquency”. Soc Forces (2012) 91(1):33–8. 10.1093/sf/sos117

[74] Saito T Shakaiteki Hikikomori: Owaranai Shishunki. Tokyo: PHP-Kenkyujo (1998).

[75] Isobe A On publication of denominational precious volumes (Jiao Pai Xi Pao Juan) in Late Ming and Early Ch’ing as seen in “Niwatazumi”. Studies of Publishing Culture in East Asia (2004) 8:187–226.

[76] Ogino T Managing categorization and social withdrawal in japan: rehabilitation process in a private support group for Hikikomorians. Int J Jpn Sociol (2004) 13:120–33. 10.1111/j.1475-6781.2004.00057.x

[77] Chan GHY Lo TW Hidden youth and the virtual world: the process of labeling and empowerment. Abingdon, Oxon: Routledge (2016). 10.4324/9781315718521

[78] Kato TA Shinfuku N Sartorius N Kanba S Are Japan’s Hikikomori and depression in young people spreading abroad? Lancet (2011) 378(9796):1070. 10.1016/S0140-6736(11)61475-X 21924990

[79] Biggs BK Vernberg EM Wu YP Social anxiety and adolescents’ friendships: the role of social withdrawal. J Early Adolesc (2012) 32(6):802–23. 10.1177/0272431611426145

[80] Tsuda H On the edge of the public space: an existentialistic contribution to the understanding and treatment of people with Hikikomori. Seishin Shinkeigaku Zasshi [Psychiatria et Neurologia Japonica] (2012) 114(10):1158–66. 23234195

[81] Hoshino Y Hikikomori to Hattatsushogai [Hikikomori and developmental disorder]. In: Dokuhon HS, editor. Naikakufu. Tokyo, Japan: Cabinet Office, Government of Japan (2011). Hikikomori Support Book], Retrieved 18 June 2014 from http://www8.cao.go.jp/youth/kenkyu/hikikomori/handbook/ua_mkj_pdf.html.

[82] Suwa M Suzuki K Hara K Watanabe H Takahashi T Family features in primary social withdrawal among young adults. Psychiatry Clin Neurosci (2003) 57:586–94. 10.1046/j.1440-1819.2003.01172.x 14629707

[83] Toivonen T Norasakkunkit V Uchida Y Unable to conform, unwilling to rebel? Youth, culture, and motivation in globalizing Japan. Front Psychology (2011) 2:1–9. 10.3389/fpsyg.2011.00207 PMC3171786 21949510

[84] Brinton M Lost in transition: youth, work, and instability in postindustrial Japan. New York, NY: Cambridge University Press (2011).

[85] Norasakkunkit V Uchida Y Psychological consequences of post industrial anomie on self and motivation among Japanese youth. J Soc Issues (2011) 67:774–86. 10.1111/j.1540-4560.2011.01727.x

[86] Norasakkunkit V Uchida Y To conform or to maintain self-consistency? Hikikomori risk in Japan and the deviation from seeking harmony. J Clin Psychol (2014) 33(10):918–35. 10.1521/jscp.2014.33.10.918

[87] Ryall J Japan’s recluses emerge and start killing. In: Telegraph. London, UK: The Telegraph (2008). June 11, Retrieved 14 May 2014 from http://www.telegraph.co.uk/comment/personal-view/3559353/Japans-recluses-emerge-and-start-killing.html.

[88] Rees P Japan: the missing million. In: BBC News World Edition. London, UK: BBC (2002). October 20, Retrieved 14 May 2014 from http://news.bbc.co.uk/2/hi/programmes/correspondent/2334893.stm

[89] Patton MQ Qualitative evaluation and research methods. 2nd ed Newbury Park: CA: Sage Publications (1990).

[90] Oiwa K NHK Yokoso—Welcome to NHK (Nippon Hikikomori Kyokai) [Anime]. Tokyo: G. Digimation (2006).

[91] Yang YX Application of ordinal regression to the research of juvenile delinquency. Master’s Thesis, Institute of Education, National Cheng Kung University: Taiwan (2009).

[92] Williams JR Gold M From delinquent behavior to official delinquency. Soc Probl (1972) 20(2):209–29. 10.2307/799615

[93] Hayes AF Introduction to mediation, moderation, and conditional process analysis. New York, NY: Guilford Press (2013).

[94] Preacher KJ Hayes AF SPSS and SAS procedures for estimating indirect effects in simple mediation models. Behav Res Methods Instrum Comput (2004) 36:717–31. 10.3758/BF03206553 15641418

[95] Bottoms A Shapland J Costello A Holmes D Muir G Towards desistance: theoretical underpinnings for an empirical study. Howard J (2004) 43(4):368–89. 10.1111/j.1468-2311.2004.00336.x

[96] Paternoster R Bachman R Kerrison E O’connell D Smith L Desistance from crime and identity: an empirical test with survival time. Crim Justice Behav (2016) 43(9):1204–24. 10.1177/0093854816651905

[97] Census and Statistics Department Revision of the Hong Kong Standard Industrial Classification. In: Hong Kong Monthly Digest of Statistics. Hong Kong: Census and Statistics Department (2008). Retrieved 8 June 2010 from http://www.statistics.gov.hk/pub/B70811FB2008XXXXB0100.pdf.

[98] Bloom N Liang J Roberts J Ying ZJ Does working from home work? Evidence from a Chinese experiment. Q J Econ (2015) 130:165–218. 10.1093/qje/qju032

[99] Wong V Youth locked in time and space? Defining features of social withdrawal and practice implications. J Soc Work Pract (2009) 23(3):337–52. 10.1080/02650530903102692

[100] Yong R Kaneko Y Hikikomori, a phenomenon of social withdrawal and isolation in young adults marked by an anomic response to coping difficulties: a qualitative study exploring individuals experiences from first- and second-person perspectives. Open j prev Med (2016) 6:1–20. 10.4236/ojpm.2016.61001

[101] Li TMH Liu L Wong PWC Withdrawal experience and possible way-outs from withdrawal behavior in young people. Qual Soc Work (2018) 17(4):537–55. 10.1177/1473325016688369

[102] Chan GHY Lo TW Quality of life of the hidden youth in Hong Kong. Appl Res Qual Life (2013) 9(4):951–69. 10.1007/s11482-013-9279-x

[103] Bushway SD Paternoster R Understanding desistance: theory testing with formal empirical models. In: MacDonald J, editor. Measuring crime and criminality: advances in criminological theory (Vol. 17). New Brunswick, NJ: Transaction (2011). p. 299–333.

[104] Bushway SD Paternoster R Desistance from crime: a review and ideas for moving forward. In: Gibson CL Krohn MD, editors. Handbook of life-course criminology. New York, NY: Springer (2013). p. 213–31. 10.1007/978-1-4614-5113-6_13

[105] Paternoster R Bushway S Desistance and the “feared self”: toward an identity theory of criminal desistance. J Crim Law Criminol (2009) 99:1103–56.

[106] Chan GHY Lo TW Hikikomori and the Internet—empowerment and disempowerment. Hong Kong: City University of Hong Kong Press (2010).

[107] Chan GHY Lo TW Do friendship and intimacy in virtual communications exist? An investigation of online friendship and intimacy in the context of hidden youth in Hong Kong. Rev Cercet Interv So (2014b) 47:117–36.

